# Surgeon and medical oncologist peer network effects on the uptake of the 21‐gene breast cancer recurrence score assay

**DOI:** 10.1002/cam4.3720

**Published:** 2021-01-16

**Authors:** Ronnie Zipkin, Andrew Schaefer, Mary Chamberlin, Tracy Onega, Alistair J. O'Malley, Erika L. Moen

**Affiliations:** ^1^ Department of Biomedical Data Science Geisel School of Medicine at Dartmouth Lebanon NH USA; ^2^ The Dartmouth Institute for Health Policy and Clinical Practice Lebanon NH USA; ^3^ Department of Medicine Geisel School of Medicine at Dartmouth Lebanon NH USA; ^4^ Department of Hematology‐Oncology Dartmouth‐Hitchcock Medical Center Lebanon NH USA; ^5^ Norris Cotton Cancer Center Dartmouth‐Hitchcock Medical Center Lebanon NH USA; ^6^ Comprehensive Breast Program Norris Cotton Cancer Center Geisel School of Medicine at Dartmouth Lebanon NH USA; ^7^ Huntsman Cancer Institute University of Utah Salt Lake City UT USA; ^8^ Department of Population Sciences University of Utah Salt Lake City UT USA; ^9^ Department of Epidemiology Geisel School of Medicine at Dartmouth Lebanon NH USA

**Keywords:** breast cancer, genetic testing, medicare, oncologists, surgeons

## Abstract

**Background:**

Drivers behind the adoption of gene expression profiling in breast cancer oncology have been shown to include exposure to physician colleagues’ use of a given genomic test. We examined adoption of the Oncotype DX 21‐gene breast cancer recurrence score assay (ODX) in the United States after its incorporation into clinical guidelines. The influence of patient‐sharing ties and co‐location with prior adopters and the role of these potential exposures across medical specialties on peers’ adoption of the test were examined.

**Methods:**

We conducted a retrospective cohort study of women with incident breast cancer using a 100% sample of fee‐for‐service Medicare enrollee claims over 2008–2011. Peer networks connecting medical oncologists and surgeons treating these patients were constructed using patient‐sharing and geographic co‐location. The impact of peer connections on the adoption of ODX by physicians and testing of patients was modeled with multivariable hierarchical regression.

**Results:**

Altogether, 156,229 women identified with incident breast cancer met criteria for cohort inclusion. A total of 7689 ODX prescribing physicians were identified. Co‐location with medical oncologists who adopted the test in the early period (2008–2009) was associated with a 1.38‐fold increase in the odds of a medical oncologist adopting ODX in 2010–2011 (95% CI = 1.04–1.83), as was co‐location with early‐adopting surgeons (odds ratio [OR] = 1.25, 95% CI = 1.00–1.58). Patients whose primary medical oncologist was linked to an early‐adopting surgeon through co‐location (OR = 1.17, 95% CI = 1.04–1.32) or both patient‐sharing and co‐location (OR = 1.17, 95% CI = 1.03–1.34) were more likely to receive ODX.

**Conclusions:**

Exposure to surgeon early adopters through peer networks and co‐location was predictive of ODX uptake by medical oncologists and testing of patients. Interventions focused on the role of surgeons in molecular testing may improve the implementation of best practices in breast cancer care.

## Graphical abstract

Surgeons have been engaged in the development of clinical algorithms involving postoperative molecular testing criteria for breast cancer patients, though promotion efforts and research into the dissemination of these technologies has primarily been focused on medical oncologists. This study demonstrates that peer influence from both early‐adopting surgeons and medical oncologists may have facilitated the uptake of the Oncotype DX recurrence score assay by their medical oncologist peers. Initiatives aimed at improving efficient and equitable incorporation of genomic and molecular innovations into breast cancer care would benefit from engaging surgeons as well as medical oncologists.

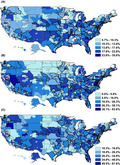

## 1 INTRODUCTION

Hormone receptor (HR)‐positive, human epidermal growth factor receptor 2 (HER2)‐negative tumors account for approximately two thirds of newly diagnosed breast cancer cases, some of which are effectively managed after surgical resection with endocrine therapy alone.^1^ Identification of patients who are unlikely to benefit from chemotherapy due to their low risk of disease recurrence is critical to spare patients undesirable side effects and sequelae associated with chemotherapy.

Several molecular genomic assays have been developed to identify the likelihood of disease recurrence in breast cancer patients, the most prominent of which is the 21‐gene recurrence score assay, Oncotype DX (ODX), administered by Genomic Health, Inc., (Redwood, CA) and made commercially available in 2004. ODX analyzes tumor gene expression to assign a recurrence score (RS) that stratifies HR‐positive, HER2‐negative patients into low, intermediate, or high‐risk categories. Patients with low and intermediate RS have been shown to have higher rates of survival and lower risk of distant recurrence, irrespective of their receiving adjuvant chemotherapy, while those with high RS are likely to benefit from chemotherapy.^2^, ^3^, ^4^ The ODX assay was first covered under Medicare in 2006 and added to guidelines for treatment of node‐negative HR‐positive, HER2‐negative breast cancers by the American Society of Clinical Oncology and National Comprehensive Cancer Network in 2007 and early 2008, respectively. Subsequent work demonstrated ODX also provides prognostic utility for patients with limited axillary lymph node involvement and has contributed to decreasing overall chemotherapy use among breast cancer patients.^5^, ^6^

While the use of ODX among Medicare‐age women receiving surgeries for HR‐positive breast cancer across the United States has grown from less than 5% of patients being tested in 2006 to as many as 20% of patients from 2012 onward, regional variation in testing patterns is still notable.^3^, ^7^, ^8^ Racial disparities in the receipt of ODX have also been seen, with a number of studies demonstrating decreased use of the test in non‐white patients.^3^, ^7^, ^8^ Along with patient characteristics, physician factors have been shown to play a role in driving the uptake of ODX, such as attitudes toward genetic testing, having completed medical training more recently, and practicing at an academic medical center.^9^, ^10^ Though ODX testing is primarily ordered by medical oncologists, as much as fifth of ODX prescriptions are ordered by surgeons.^10^

In addition to exposure through the publication of clinical trial results, updating of clinical guidelines, and new regulatory approvals, medical oncologists have expressed that the adoption of a genetic testing modality is also influenced by the use of a test by their colleagues.^11^, ^12^ One approach to identifying how peer influence impacts the uptake of new treatments is the measurement of exposure to adopters via relationships in patient‐sharing networks, which frequently correspond to professional relationships in clinical practice.^13^ In breast cancer care, the effect of peer exposure via patient‐sharing networks on adoption has been demonstrated for new surgical and radiologic methods, as well as for the uptake of ODX among medical oncologists.^14^, ^15^, ^16^ However, social networks defined by connections that are limited to a single type of relationship can often be an oversimplification of reality. By examining peer exposure through a co‐location network in addition to a patient‐sharing network, it may be possible to capture additional relationships not detectible through patient‐sharing alone. The premise for examining the role of co‐location in the diffusion of medical innovations is supported by previous work demonstrating that geographic proximity to prior adopters can serve as a proxy for a physician's peer exposure to new medical technologies beyond that explained by marketing efforts, professional events, patient requests, or characteristics intrinsic to adopters.^17^, ^18^

We hypothesized that co‐location would capture important mechanisms of peer influence among cancer specialists beyond what has been observed within patient‐sharing networks. Given that ODX is sometimes ordered by surgeons, we further specify whether patient‐sharing or co‐location with an early adopting surgeon was present. Our objective was to assess the influence of peer network exposure––defined using patient‐sharing and co‐location––on physician adoption and patient receipt of the ODX test, adjusting for other patient, physician, and regional characteristics. As the frequency of ODX testing in the Medicare population had previously been shown to vary by region, we also analyzed regional variation and how the extent to which surgeons comprise ODX prescribers in a region might impact ODX use.

## 2 METHODS

### 2.1 Study population

We conducted a retrospective cohort study of breast cancer patients in a 100% sample of Medicare fee‐for‐service beneficiaries from 2007 to 2012. Women with incident breast cancer and treating physicians were identified using the Centers for Medicare and Medicaid Services (CMS) Master Beneficiary Summary, Medicare Provider Analysis and Review, Carrier, and Outpatient Services files. Approval was obtained from the institutional review board at Dartmouth College.

We identified incident breast cancer cases and respective diagnosis dates among female beneficiaries aged 65–99 using the biopsy and surgery claims algorithms of Bronson et al.^19^ Procedure codes accounting for trends over the 2006–2010 period toward increased use of reconstructive surgical approaches were included, based on patterns evident in the Dartmouth Atlas Health care Coding Trends (Table S1).^20^ Patients were included if they were continuously enrolled in Parts A and B over the year prior to and following their date of diagnosis. Then, patients unlikely to be candidates for ODX were excluded from the cohort by the method of Su et al., specifically patients not undergoing breast cancer surgery, those with stage IV disease, and those receiving trastuzumab.^7^ This method implements the validated claims‐based algorithm of Smith et al., of which we applied the parameter estimates of step one (Model to Predict Stage IV Disease) with a probability cutpoint of 0.15 in order to exclude patients with advanced stage disease who are unlikely to receive the test.^21^ Based on their date of diagnosis, the remaining patients were then divided into being considered treated in the “early” (2008–2009) or “late” (2010–2011) time periods, ranging from the initial publishing of guidelines supporting ODX use to shortly thereafter, respectively, for comparability to previous work by Rotter et al.^16^

### 2.2 Construction of physician peer patient‐sharing and co‐location networks

Physicians were included in the networks if they had claims treating any incident breast cancer patient, irrespective of predicted stage or treatment type, in the 3 months prior to and 12 months following a patient's diagnosis date. Connections between physicians were identified by either shared patients or co‐location. Physicians were linked within the patient‐sharing network if they shared two or more patients, a threshold previously shown sufficient to detect professional relationships and preserve network structures.^13^, ^22^ The co‐location network was based on ZIP code tabulation areas (ZCTAs) of Part B claims. Physicians were considered connected within the co‐location network if they had five or more breast cancer‐related claims in the same ZCTA during the early period, allowing for physicians to appear in multiple ZCTAs. We took this approach to enrich for physicians who are engaged in active practice at each location, and to account for possible interactions at different facilities or institutions. We further characterized each physician dyad as being either (a) connected through shared patients and not co‐located, (b) connected through co‐location without shared patients, (c) connected through both, or (d) not connected.

### 2.3 Study variables

Cohort patient age and race were obtained from the CMS Master Beneficiary Summary File. We identified patients who were treated at a teaching hospital and had encounter claims with two or more medical oncologists. Based on patient ZCTA of residence, we obtained the area‐level measures of rurality and poverty by linking to the U.S. Department of Agriculture Economic Research Service Rural‐Urban Commuting Area primary designations^23^ and U.S. Census Small Area Income and Poverty Estimates.^24^ Patient and physician ZCTAs were used to assign individuals to Dartmouth Atlas hospital referral regions (HRRs), which were linked to Atlas Hospital and Physician Capacity Measures^25^ and the CMS Geographic Variation Public Use File.^26^

Physician specialty and gender were queried by National Provider Identifier (NPI) using the CMS Physician Compare^27^ and National Plan and Provider Enumeration System^28^ National Downloadable Files, as well as by the National Claims History data accessed via the CMS Carrier File. We calculated medical oncologist patient volume as the sum of unique cohort patients attributed to each medical oncologist within each time period. Primary medical oncologists were assigned to patients by a modification of the method of Keating et al. hierarchically, based on whether they (a) prescribed ODX to the patient or (b) were the medical oncologist with whom the patient had the most visits.^29^ In the event of a tie for (2), the medical oncologist who saw the patient closest to the date of diagnosis was assigned to the patient.

Our primary outcome variable was physician adoption of ODX. Use of the ODX test was captured by a modification of the method of Dinan et al.^30^ Briefly, Part B claims were identified that contained: (a) procedure code 84999 (Chemistry Procedures), (b) NPI = 1215003603 (Genomic Health, Inc.), and (c) a cost not equal to $3104 (representing the Oncotype DX colon cancer recurrence test). Per Rotter et al., “early adoption” by a physician was also defined as prescribing the ODX test to any breast cancer patient at least once in the early time period (2008–9), and “late adoption” among those having seen at least one cohort patient in the early period but not having previously ordered the test was defined as prescribing the ODX test at least once during the late period (2010–11).^16^ Medical oncologists who had prescribed the assay in the early period were excluded from physician‐level analyses of adoption in the late period. We also examined patient receipt of the test to allow for better adjustment of patient‐level characteristics.

We calculated several measures of exposure and connectedness for each medical oncologist based on their position within the patient‐sharing and co‐location networks described above. We considered a medical oncologist in the late period “exposed” if they were connected to an early adopting medical oncologist or surgeon through patient‐sharing, co‐location, or both. For each medical oncologist, we measured the number of patient‐sharing connections to other medical oncologists and to surgeons (analogs of degree centrality, a common network measure used to identify highly connected individuals).

### 2.4 Regional ODX practice patterns

To describe and visualize the regional patterns in ODX use, cohort patients, and ODX‐prescribing physicians across the study period were aggregated by HRR. To differentiate areas by health care market size, we stratified HRRs into those above or below the 75th‐percentile of total cohort patients treated over 2008–2011, referred to as “upper” or “lower” stratum, respectively. Cohort and physician characteristics were mapped to HRR and visualized using ArcGIS (Version 10.7.1; Environmental Systems Research Institute, Redlands, CA).

### 2.5 Statistical methods

Bivariate associations between study variables and receipt of the ODX test among patients with assigned medical oncologists were first assessed by two‐sided Pearson's chi‐squared test for independence for categorical variables or Mann–Whitney U‐tests for medians. Adoption of the ODX test among physicians treating at least one cohort patient and receipt of the test by cohort patients were modeled as binary outcomes using multilevel logistic regression with a random effect for physician HRR or for both the patient's primary medical oncologist and HRR of residence, respectively. Recognizing that high volume physicians are more likely to have an earlier encounter with an eligible patient, we also modeled ODX adoption in each time period stratified by the volume of cohort patients seen by each medical oncologist. Pearson's correlation test coefficients and *p*‐values were calculated for HRR‐level analyses.

## 3 RESULTS

Altogether, 302,826 women with incident breast cancer were identified in Medicare claims over the 2008–2011 period. Of the final cohort patients with an assigned medical oncologist (N = 156,229), 18,244 (11.7%) received the ODX test (Table 1). Patients who received the ODX test over the study period were more often younger, white, seen by two or more medical oncologists, and rural‐residing, as compared to patients not receiving the test. Prescribers of ODX (N = 7689) were composed of 73.6% medical oncologists and 21.1% surgeons. Patient volume was strongly associated with adoption among medical oncologists in both the early and late time periods (Table 2 and Table S2).

**TABLE 1:** Patient characteristics among cohort members with an assigned primary medical oncologist (2008–2011)

		No ODX N = 137,985	ODX N = 18,244	*p* ^a^
Age at diagnosis	65–69	34,696 (25.1%)	7560 (41.4%)	<0.001
	70–75	34,043 (24.7%)	6165 (33.8%)	—
	76–79	30,008 (21.7%)	3190 (17.5%)	—
	80+	39,238 (28.4%)	1329 (7.3%)	—
Race	White	123,662 (89.6%)	16,735 (91.7%)	<0.001
	Black	10,050 (7.3%)	1005 (5.5%)	—
	Other	4273 (3.1%)	504 (2.8%)	—
Treated at Teaching Hospital		33,609 (24.4%)	4551 (24.9%)	0.084
Visited Two or More Medical Oncologists		39,743 (28.8%)	6274 (35.4%)	<0.001
Region	Northeast	26,022 (18.9%)	3236 (17.7%)	<0.001
	Midwest	34,105 (24.4%)	4982 (25.8%)	—
	South	53,429 (38.7%)	7332 (40.2%)	—
	West	24,429 (17.7%)	2968 (16.3%)	—
Rural		28,759 (20.8%)	4038 (22.1%)	<0.001

Abbreviations: ODX, Oncotype DX.

^a^ Two‐sided Chi‐squared test *p*‐values

**TABLE 2:** Characteristics of medical oncologists in the late period (2010–2011)

		Non‐adopter N = 2505	Adopter N = 1260	*p* ^a^
Gender	Female	587 (23.4%)	318 (25.2%)	0.24
Patient volume	1–4	1210 (48.3%)	185 (14.7%)	<0.001
	5–9	575 (23.0%)	351 (28.9%)	—
	10–19	412 (16.4%)	471 (37.4%)	—
	20+	308 (12.3%)	253 (20.1%)	—
Number of Patient‐Sharing Ties to Medical Oncologists (median [IQR])		5 [2,11]	9 [6,14]	<0.001
Number of Patient‐Sharing Ties to Surgeons (median [IQR])		0 [0,2]	1 [0,4]	<0.001
Connection to Early Adopter Medical Oncologist	None	651 (26.0%)	151 (12.0%)	<0.001
	Co‐Location	1039 (41.5%)	571 (45.3%)	—
	Patient‐Sharing	76 (3.0%)	29 (2.3%)	—
	Both	739 (29.5%)	509 (40.4%)	—
Connection to Early Adopter Surgeon	None	985 (39.3%)	302 (24.0%)	<0.001
	Co‐Location	1169 (36.7%)	713 (56.6%)	—
	Patient‐Sharing	28 (1.1%)	19 (1.5%)	—
	Both	323 (12.9%)	226 (17.9%)	—

Abbreviations: IQR, interquartile range.

^a^ Two‐sided Chi‐squared test *p*‐values for categorical variables, Mann–Whitney U‐test *p*‐values for medians

Adjusted physician‐level analyses showed medical oncologists were more likely to adopt in the early time period if they were female, treated a higher volume of breast cancer patients, had more patient‐sharing ties to surgeons, and had fewer patient‐sharing ties to medical oncologists (Table S3). Patient‐level factors associated with receiving ODX during the early time period included younger age, white race, and being seen by two or more medical oncologists (Table S4).

To test the hypothesis that exposure to ODX testing through prior peer network connections would increase a physician's likelihood of adopting the test, we examined how connections to early adopters impacted uptake of ODX among medical oncologists in the late time period (Table 3). We found that co‐location with an early adopting medical oncologist increased the odds of adoption by 1.38‐fold (95% confidence interval [CI] = 1.04–1.83). Medical oncologists were also more likely to adopt ODX if they were connected to an early adopting surgeon through co‐location (odds ratio [OR] = 1.25, 95% CI = 1.00–1.58) and patient‐sharing (OR = 1.85, 95% CI (0.93,3.67), but these relationships did not achieve statistical significance at the *p* < 0.05 level.

**TABLE 3:** Medical oncologist‐level model of the association between 2010 and 2011 adoption of oncotype DX with geographic and patient‐sharing connections to early adopters^a^

	N = 3765	Crude OR (95% CI)	*p*	Adjusted^b^ OR (95% CI)	*p*
Gender (Ref: Male)	Female	1.11 (0.94,1.31)	0.20	1.11 (0.93,1.33)	0.24
Patient Volume (Ref: 1–4)	5–9	4.00 (3.24,4.93)	<0.001	4.19 (3.35,5.25)	<0.001
	10–19	7.49 (6.07,9.24)	<0.001	8.09 (6.30,10.40)	<0.001
	20+	5.40 (4.27,6.82)	<0.001	6.50 (4.61,9.17)	<0.001
Number of Patient‐Sharing Ties to Medical Oncologists		1.04 (1.04,1.05)	<0.001	0.96 (0.95,0.98)	<0.001
Number of Patient‐Sharing Ties to Surgeons		1.15 (1.11,1.18)	<0.001	1.08 (1.04,1.13)	<0.001
Connection to Early Adopter Medical Oncologist (Ref: None)	Co‐Location	2.37 (1.92,2.93)	<0.001	1.38 (1.04,1.83)	0.03
	Patient‐Sharing	1.66 (1.03,2.67)	0.03	0.98 (0.58,1.67)	0.95
	Both	2.97 (2.39,3.68)	<0.001	1.19 (0.88,1.63)	0.25
Connection to Early Adopter Surgeon (Ref: None)	Co‐Location	2.02 (1.72,2.39)	<0.001	1.25 (1.00,1.58)	0.05
	Patient‐Sharing	2.27 (1.23,4.22)	<0.001	1.85 (0.93,3.67)	0.07
	Both	2.31 (1.85,2.89)	<0.001	1.13 (0.85,1.52)	0.39

Abbreviations: CI, confidence interval; HRR, hospital referral region; OR, odds ratio; RUCA, rural‐urban commuting area.

^a^ Mixed‐effects logistic regression of adoption by the end of 2011, Random effect for HRR.

^b^ Also adjusted for census region, RUCA designation, area poverty, as well as HRR Medicare Advantage participation, minority population size, and *per capita* physicians and medical oncologists.

To examine the effects of physician peer influence while accounting for patient‐level variables, we then modeled its impact on patient receipt of ODX over 2010–2011 (Table 4). As we also observed in the early time period, younger age at diagnosis, having a female primary medical oncologist, and being seen by two or more medical oncologists were associated with an increased likelihood of receiving the test. Black patients were over 30% less likely to receive the test, and other non‐white patients were 13% less likely to be tested. A patient's primary medical oncologist's connection to an early adopter surgeon by co‐location was positively associated with the patient receiving ODX (OR = 1.17, 95% CI = 1.04–1.32), as well as was combined co‐location and patient‐sharing (OR = 1.17, 95% CI = 1.03–1.34). Interestingly, in adjusted analyses of both medical oncologist‐level adoption and patient‐level receipt of ODX, a medical oncologist's number of patient‐sharing ties to surgeons was positively associated with ODX use, while the number of ties to other medical oncologists was consistently negatively associated with use of the test.

**TABLE 4:** Patient‐level model of oncotype DX receipt in 2010–2011^a^

	N = 76,359	Crude OR (95% CI)	*p*	Adjusted OR (95% CI)	*p*
Patient predictors					
Age (Ref: 65–69)	70–74	0.87 (0.82,0.92)	<0.001	0.87 (0.83,0.92)	<0.001
	75–79	0.52 (0.49,0.55)	<0.001	0.52 (0.49,0.55)	<0.001
	80+	0.16 (0.15,0.17)	<0.001	0.16 (0.15,0.17)	<0.001
Race (Ref: White)	Black	0.69 (0.62,0.76)	<0.001	0.62 (0.56,0.68)	<0.001
	Other	0.92 (0.80,1.05)	0.19	0.87 (0.76,1.00)	0.05
Treated at Teaching Hospital		1.07 (1.01,1.15)	0.03	1.00 (0.94,1.07)	0.99
Visited Two or More Medical Oncologists (Ref: 1)		1.28 (1.22,1.35)	<0.001	1.14 (1.09,1.20)	<0.001
Rural (Ref: Urban)		1.12 (1.05,1.19)	<0.001	1.07 (1.00,1.14)	0.05
Area Poverty	≥ 20%	0.99 (0.92,1.06)	0.71	1.00 (0.92,1.08)	0.97
Primary medical oncologist predictors					
Gender (Ref: Male)	Female	1.09 (1.02,1.17)	0.02	1.09 (1.01,1.17)	0.02
Patient Volume (Ref: 1–4)	5–9	1.19 (1.02,1.40)	0.03	1.15 (0.97,1.36)	0.10
	10–19	1.18 (1.02,1.37)	0.03	1.09 (0.93,1.28)	0.28
	20+	1.04 (0.90,1.21)	0.59	0.92 (0.77,1.10)	0.36
Number of Patient‐Sharing Ties to Medical Oncologists		1.00 (1.00,1.00)	0.35	1.00 (0.99,1.00)	0.23
Number of Patient‐Sharing Ties to Surgeons		1.00 (0.99,1.01)	0.51	1.00 (0.99,1.02)	0.65
Connection to Early Adopter Medical Oncologist (Ref: None)	Co‐Location	1.22 (1.04,1.43)	0.007	1.16 (0.96,1.40)	0.13
	Patient‐Sharing	1.15 (0.84,1.57)	0.39	1.14 (0.82,1.58)	0.44
	Both	1.23 (1.06,1.44)	0.01	1.18 (0.97)	0.09
Connection to Early Adopter Surgeon (Ref: None)	Co‐Location	1.19 (1.08,1.31)	<0.001	1.17 (1.04,1.32)	0.008
	Patient‐Sharing	0.95 (0.67,1.35)	0.76	0.93 (0.65,1.35)	0.71
	Both	1.16 (1.05,1.30)	0.004	1.17 (1.03,1.34)	0.02
Region (Ref: Northeast)	Midwest	1.05 (0.93,1.18)	0.47	1.04 (0.92,1.18)	0.54
	South	1.03 (0.92,1.15)	0.58	0.96 (0.85,1.08)	0.51
	West	0.91 (0.80,1.04)	0.15	0.90 (0.78,1.03)	0.13
HRR predictors					
% with Medicare Advantage	Top 50^th^ Percentile	0.88 (0.82,0.95)	0.001	0.90 (0.83,0.97)	0.008
% Black	Top Quintile	1.13 (1.04,1.24)	0.005	1.11 (1.01,1.23)	0.03
% Hispanic	Top Quintile	0.98 (0.90,1.07)	0.61	1.06 (0.95,1.18)	0.32
% Other Race	Top Quintile	0.96 (0.88,1.04)	0.29	0.97 (0.88,1.08)	0.56
Physicians per capita	Bottom Quintile	0.95 (0.85,1.07)	0.40	1.01 (0.89,1.14)	0.91
Medical Oncologists per capita	Bottom Quintile	0.89 (0.79,0.99)	0.03	0.91 (0.80,1.03)	0.14

Abbreviations: CI, confidence interval; HRR, hospital referral region; OR, odds ratio.

^a^ Mixed‐effects logistic regression, Random effects for primary medical oncologist and HRR.

As high‐volume medical oncologists are more likely to have an earlier encounter with a patient eligible for ODX compared with low volume physicians, we stratified medical oncologists by patient volume (Tables S5 and S6). We found the effects of exposure to ODX through the peer network were most pronounced among low volume medical oncologists but were attenuated among those with higher patient volume. Among low volume medical oncologists, sharing patients with an early‐adopting surgeon corresponded to a 3.86‐fold increase (95% CI = 1.08–13.86), co‐location with an early adopting medical oncologist corresponded to a 2.12‐fold increase (95% CI = 1.24–3.62), and combined co‐location and sharing patients with an early‐adopting medical oncologist corresponded to a 2.85‐fold increase (95% CI = 1.50–5.42) in the likelihood of adopting ODX.

### 3.1 Geographic variation in ODX and breast cancer care

**FIGURE 1:**
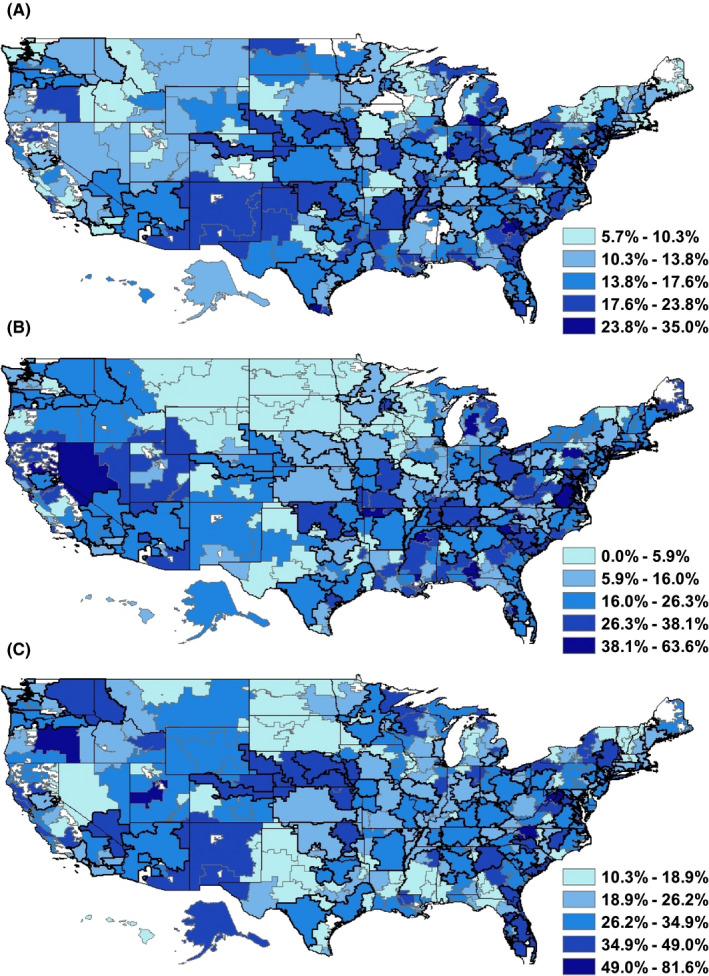
Regional Variation in Oncotype DX Practice Patterns (2008–2011). Numbers indicate (A) the percent of total cohort patients receiving the Oncotype DX (ODX) test, (B) the percent of ODX‐prescribing physicians who were surgeons, and (C) the percent of cohort patients treated by two or more medical oncologists by hospital referral region (HRR). The boundaries of HRRs were bolded if they were above the 75th‐percentile of total cohort patients treated. White areas indicate HRRs where proportions would have involved patient counts fewer than 11

Finally, we explored regional variation of ODX practices across HRRs. Rates of ODX receipt among patients by HRR (Figure 1A) were consistent with previous findings by Lynch and colleagues.^8^ We stratified the HRRs into an upper and lower strata based on cohort patient volume over 2008–2011; HRRs in upper stratum had a median of 1090 patients (interquartile range [IQR] = 867–1436) and those in the lower stratum had a median of 284 patients (IQR = 179–426). The median number of ODX prescribers per HRR in the lower stratum was 13 (IQR = 7–19), whereas the upper stratum had many more prescribers (median = 51, IQR = 38–74). Given the observed importance of surgeons’ peer network influence on the adoption and diffusion of the test among medical oncologists, we assessed the proportion of ODX prescribers who were surgeons in a given HRR (Figure 1B). This was positively correlated with the proportion of patients receiving the test in the lower stratum (*ρ* = 0.234, *p* < 0.001) and upper stratum (*ρ* = 0.0402, *p* < 0.001), indicating the potential importance of surgeon prescribers, especially in smaller health care markets. We also observe considerable variability across HRRs in the percent of cohort patients treated by two or more medical oncologists (Figure 1C), which we had observed to be a significant predictor of ODX testing in our patient‐level models. The percent of patients having been seen by two or more medical oncologists was positively correlated with the percent of patients receiving ODX in the upper stratum (*ρ* = 0.257, *p* = 0.02).

## 4 DISCUSSION

Using claims from the full nationwide Medicare beneficiary data over 2008–2011, we identified a number of patient, physician, and regional attributes associated with the use and dissemination of ODX. Compared with previously reported national Medicare and Surveillance, Epidemiology, and End Results (SEER)‐Medicare data, we found similar regional patterns of ODX testing and a comparable proportion of prescribers who were medical oncologists (73.6%), though the proportion of prescribers who were surgeons in our national cohort (21.1%) appears slightly greater than that reported for SEER‐Medicare.^9^, ^10^ Consistent with other studies, we found that younger patient age, white race, and having a female medical oncologist were positively associated with receiving ODX.^7^, ^8^, ^10^, ^16^

Patient volume was a strong predictor of ODX adoption in all physician‐level models. Hospital and physician‐level patient volume have been positively associated with best practices and improved outcomes in breast cancer care.^31^, ^32^, ^33^ Previous work has also indicated that physicians with larger patient numbers and prescribing volumes are more likely to adopt new treatments.^34^

Our results provide additional evidence of peer influence by early adopting medical oncologists through patient‐sharing as seen in recent analyses of ODX adoption,^16^ but our works adds to this literature in several ways. First, we found that links between surgeons and medical oncologists identified through co‐location capture an important aspect of innovation diffusion unmeasured when connections are only identified through patient‐sharing. This finding is also consistent with recent work showing expanded prescribing of new cancer drugs geographically clustered near physicians involved with clinical trials related to these treatments.^35^ Second, when stratifying by patient volume, we observed the strongest peer effects for low volume physicians. Low volume physicians may be more reliant on peers for changing their practice patterns to be consistent with new guidelines, a hypothesis that could be tested in future work. Third, by considering patient‐sharing across specialties, we found that early‐adopting surgeons may have played an important role in the diffusion of ODX among medical oncologists. The ordering of ODX by surgeons has been considered as a means to reduce delays in initiation of treatment following ODX testing.^36^ Surveys on the utilization of genomic tests in cancer care typically focus on medical oncologists, although some also include surgical oncologists^9^, ^32^, ^33^; as such, the limited knowledge available on overp'all surgeon perspectives in this realm may warrant further study. Previous work has suggested that surgeon‐initiated ODX testing protocols are unlikely to lead to inappropriate overuse of the test.^37^ Since the percent of patients receiving ODX in an HRR was positively correlated with the percent of surgeon prescribers, it may be the case that health systems with surgeon‐initiated testing protocols facilitated efficient and appropriate uptake of the test. Increased adoption among medical oncologists co‐located with early adopters may also be explained by participation in a molecular, precision medicine, or other multidisciplinary tumor board, where discussion regarding similar patients may offer opportunities for information exchange and the updating of a prior non‐adopter's testing protocols.

Our study also uncovered an association between seeing two or more medical oncologists and ODX testing. While we cannot say using administrative data whether the patient sought out a second opinion, an earlier study found an association between seeking second opinions and ODX testing, particularly among patients receiving intermediate RS results.^38^ Factors such as patient education, physician‐patient communication, and clinical cases requiring significant patient input on decision making can motivate the use of second opinions in breast cancer oncology.^38^, ^39^ Informed patients advocating for themselves, as has been speculated might play a role in increasing the rates of hereditary BRCA testing and risk‐reducing mastectomy,^40^, ^41^ could represent a driver of second opinion‐seeking, physician ODX adoption, and future prescribing by the physician of ODX for other patients. It is also possible that patients who are high utilizers of health care may be more likely to visit two or more medical oncologists and more likely to receive ODX testing. Our findings support further exploration of patterns of second opinion‐seeking relative to patient access to new medical technologies.

Patient‐sharing networks are a promising approach for exploring how the relationships between physicians impacts health care utilization, costs, and quality.^29^, ^42^, ^43^ Strengths of our study design include our use of a national breast cancer physician network based on patient‐sharing and co‐location. A national network reduces biases that may be introduced when the network is constrained by a geographic boundary (e.g., state), which can impact the accuracy of network measures.^44^ Many cancer provider network studies are limited to small areas or subsamples of the SEER‐Medicare‐linked database, which has the benefit of additional clinical and demographic details but may underrepresent certain types of patients, such as patients who are rural‐residing.^45^ Our work highlights the added insight that can be gleaned from these networks by integrating networks that capture relationships through geographic proximity.^46^ While previous work has distinguished within‐hospital from between‐hospital patient‐sharing ties,^47^ this study defines mutually exclusive ties based on whether physicians share patients, are co‐located, or both. Our results provide evidence that co‐location with adopters is an important exposure to consider when examining adoption of genomic testing in breast cancer.

Our study has several limitations. First, we relied on an established claims‐based algorithm to identify incident breast cancer patients for our study cohort and as such we are limited by the sensitivity and specificity of this methodology. Second, the number of patients shared by any pair of physicians likely represents a lower bound of all shared patients, since they may also share breast cancer patients not enrolled in Medicare or not identified in claims. Third, although we implemented an established algorithm to exclude patients unlikely to be eligible for ODX testing, stage and other clinicopathological characteristics are not directly defined in claims data but impact a patient's eligibility for ODX. Fourth, we did not examine the uptake of other molecular assays by physicians, whether patients received these, or what associations might have existed between the use of other tests and ODX, as has been examined by others.^8^, ^9^ Fifth, our study does not directly account for information routes exogenous to the identified peer networks, such as publication dates of individual clinical studies, periods specific to physician‐oriented or direct‐to‐patient advertising efforts, health policy modifications, or health facility administrative changes that may have influenced testing rates; however, these may in some cases coincide with or be detected as co‐location exposure, even if co‐located physicians do not personally interact. Sixth, our results may not be generalizable to populations outside of the Medicare fee‐for‐service population, and we were unable to capture ODX prescribing for patients under 65 years of age, which may have impacted the early adopter status of physicians, especially those who saw fewer Medicare patients. Finally, although we examined the association between peer use of ODX in the early time period and subsequent adoption in the late time period, our findings are observational and should not be considered causal.

Overall, our study further supports an important role for physician peer networks in the adoption and diffusion of novel treatment and molecular testing modalities in cancer care, while also highlighting the role surgeons may play in this process. We expect that future work defining peer networks through co‐location and patient‐sharing ties will provide important insight into the role of peer influence on the diffusion of cancer care innovations. This work demonstrates the potential for the wider use of claims data to study geographic variation in clinical practice, the diffusion of innovations across provider networks, and disparities in patient access to the newest medical technologies.

## AUTHOR CONTRIBUTIONS

Ronnie Zipkin: Conceptualization, data curation, formal analysis, investigation, methodology, software, visualization, and writing––original draft. Andrew Schaefer: Data curation, investigation, methodology, resources, software, and visualization. Mary Chamberlin: Conceptualization, writing––review, and editing. Tracy Onega: Conceptualization, writing––review, and editing. Alistair J. O'Malley: Conceptualization, methodology, supervision, writing––review, and editing. Erika L. Moen: Conceptualization, data curation, formal analysis, funding acquisition, investigation, methodology, project administration, resources, software, supervision, and writing––original draft.

## Supporting information

TableS1‐S6

## Data Availability

The administrative data underlying this article were provided by the Centers for Medicare and Medicaid Service (CMS) under Data Use Agreement #52234. These data contain patient protected health information and cannot be shared outside of the members of this study, as specified by the agreement with the Research Data Assistance Center at CMS. Physician Compare, National Provider Identifier, and Geographic Variation public use data used in this study are available through CMS: [https://download.cms.gov/nppes/NPI_Files.html, https://data.medicare.gov/data/physician‐compare, https://www.cms.gov/Research‐Statistics‐Data‐and‐Systems/Statistics‐Trends‐and‐Reports/Medicare‐Geographic‐Variation/GV_PUF]. Medicare coding trends and hospital and physician capacity measures used herein are available through the Dartmouth Atlas of Healthcare. The datasets were derived from sources in the public domain: [https://atlasdata.dartmouth.edu/downloads/supplemental#coding_trends, https://atlasdata.dartmouth.edu/downloads/general#hospital_capacity]. Small Area Income and Poverty Estimates were obtained through US Census Bureau public domain data: [https://www.census.gov/programs‐surveys/saipe.html]. Rural‐Urban Commuting Area designations were made available to the public domain by the US Department of Agriculture Economic Service: [https://www.ers.usda.gov/data‐products/rural‐urban‐commuting‐area‐codes/].
