# Supplementary material for: Surgeon and medical oncologist peer network effects on the uptake of the 21‐gene breast cancer recurrence score assay
Source: Cancer Med. 2021 Jan 16;10(4):1253–63. doi: 10.1002/cam4.3720 (PMC7926024; doi:10.1002/cam4.3720)
Supplement: Supplementary file 1 — TableS1‐S6 [file CAM4-10-1253-s001.pdf]

**Supporting Table 1**. Medical Codes Used to Identify Breast Cancer Diagnoses, Surgeries, and Biopsies in Medicare Claims

| Measure | Codes | |
|---|---|---|
| Diagnosis | ICD9 diagnosis | 174, 1740, 1741, 1742, 1743, 1744, 1745, 1756, 1748, 1749, 2330 |
| Surgery | ICD9 procedure | 344, 857, 3401, 4029, 403, 4050, 4051, 4059, 409, 8520, 8521, 8522, 8523, 8531, 8532, 8533, 8534, 8535, 8536, 8541, 8542, 8543, 8544, 8546, 8547, 8570, 8524, 8525, 8553, 8554, 8555, 856, 8571, 8572, 8573, 8574, 8575, 8576, 8581, 8582, 8583, 8584, 8585, 8586, 8587, 8589, 8591, 8592, 8593, 8594, 8595, 8596, 8599 |
| | HCPCS CPT | 19112, 19120, 19125, 19126, 19160, 19162, 19180, 19182, 19200, 19220, 19240, 19260, 19271, 19272, 19301, 19302, 19303, 19304, 19305, 19306, 19307, 38505, 38525, 38470, 38745, 15777, 19340, 19342, 19350, 19355, 19357, 19361, 19364, 19366, 19367, 19368, 19369, 19380, 38500, 38792, 78195, 78800, 78801 |
| Biopsy | ICD9 procedure | 851, 8511, 8512, 8519, 4023, 8321 |
| | HCPCS CPT | 19081, 19082, 19083, 19084, 19085, 19086, 19100, 19101, 19102, 19103, 1911, 19281, 19282, 19283, 19284, 19285, 19286, 19287, 19288, 38792, 77031, 77032, 88305, 88309, 10222 76942, 19295, 77012 |

Abbreviations: ICD9 = International Classification of Diseases, 9th Revision; HCPCS = Healthcare Common Procedure Coding System; CPT = Common Procedural Terminology

**Supporting Table 2.** Characteristics of Medical Oncologists in the Early Period (2008-2009)

| | | Non-Adopter N=4,740 | Adopter N=4,355 | P[a] |
|---|---|---|---|---|
| Gender | Female | 1,160 (24.5%) | 1,303 (29.9%) | <0.001 |
| Patient Volume | 1-4 | 2,307 (48.7%) | 373 (8.6%) | <0.001 |
| | 5-9 | 1,023 (21.6%) | 782 (18.0%) | - |
| | 10-19 | 412 (16.4%) | 471 (37.4%) | - |
| | 20+ | 308 (12.3%) | 253 (20.1%) | - |
| Number of Patient-Sharing Ties to Medical Oncologists (median [IQR]) | | 5 [2,11] | 9 [6,14] | <0.001 |
| Number of Patient-Sharing Ties to Surgeons (median [IQR]) | | 0 [0,2] | 1 [0,4] | <0.001 |
| Region | Northeast | 1,160 (24.5%) | 874 (20.1%) | <0.001 |
| | Midwest | 1,033 (21.8%) | 1,077 (24.7%) | - |
| | South | 1,651 (34.8%) | 1,632 (37.5%) | - |
| | West | 896 (18.9%) | 772 (17.7%) | - |
| Rural | | 813 (17.2%) | 794 (18.2%) | 0.18 |

[a] Two-sided Chi-squared test P-values for categorical variables, Mann-Whitney U-test P-values for medians

Abbreviations: IQR = interquartile range

**Supporting Table 3**. Medical Oncologist-Level Model of Oncotype DX Adoption in 2008-2009[a]

| | N=9,095 | Crude OR (95% CI) | P | Adjusted OR (95% CI) | P |
|---|---|---|---|---|---|
| Gender (Ref: Male) | Female | 1.38 (1.25,1.52) | <0.001 | 1.35 (1.21,1.51) | <0.001 |
| Patient Volume (Ref: 1-4) | 5-9 | 4.78 (4.12,5.55) | <0.001 | 4.91 (4.21,5.71) | <0.001 |
| | 10-19 | 11.74 (10.19,13.52) | <0.001 | 11.35 (9.72,13.26) | <0.001 |
| | 20+ | 18.96 (16.27,22.1) | <0.001 | 16.71 (13.71,20.36) | <0.001 |
| Number of Patient-Sharing Ties to Medical Oncologists | | 1.11 (1.10,1.12) | <0.001 | 0.94 (0.93,0.96) | <0.001 |
| Number of Patient-Sharing Ties to Surgeons | | 1.31 (1.28,1.33) | <0.001 | 1.11 (1.08,1.13) | <0.001 |
| Region (Ref: Northeast) | Midwest | 1.37 (1.16,1.63) | <0.001 | 1.14 (0.95,1.38) | 0.15 |
| | South | 1.34 (1.15,1.57) | <0.001 | 1.08 (0.91,1.29) | 0.39 |
| | West | 1.18 (0.98,1.41) | 0.08 | 1.02 (0.82,1.26) | 0.88 |
| Rural (Ref: Urban) | | 1.00 (0.89,1.13) | 0.97 | 0.92 (0.80,1.06) | 0.23 |
| Area Poverty | ≥ 20% | 0.95 (0.80,1.13) | 0.56 | 1.03 (0.84,1.25) | 0.79 |
| Hospital Referral Region | | | | | |
| % with Medicare Advantage | Top 50th Percentile | 0.82 (0.73,0.93) | 0.001 | 0.97 (0.85,1.11) | 0.66 |
| % Black | Top Quintile | 0.96 (0.83,1.11) | 0.56 | 1.00 (0.84,1.18) | 0.98 |
| % Hispanic | Top Quintile | 0.78 (0.68,0.90) | <0.001 | 1.02 (0.85,1.22) | 0.86 |
| % Other Race | Top Quintile | 0.79 (0.69,0.90) | <0.001 | 0.97 (0.82,1.16) | 0.75 |
| Physicians per capita | Bottom Quintile | 1.21 (1.01,1.44) | 0.03 | 1.13 (0.92,1.39) | 0.25 |
| Medical Oncologists per capita | Bottom Quintile | 1.23 (1.03,1.47) | 0.02 | 0.92 (0.75,1.13) | 0.42 |

[a] Mixed-effects logistic regression of adoption by the end of 2009, Random effect for HRR

Abbreviations: CI = confidence interval, HRR = hospital referral region, OR = odds ratio

**Supporting Table 4**. Patient-Level Model of Oncotype DX Receipt in 2008-2009 [a]

| | N=79,995 | Crude OR (95% CI) | P | Adjusted OR (95% CI) | P |
|---|---|---|---|---|---|
| Patient Predictors | | | | | |
| Age (Ref: 65-69) | 70-74 | 0.77 (0.72,0.81) | <0.001 | 0.77 (0.72,0.82) | <0.001 |
| | 75-79 | 0.43 (0.40,0.46) | <0.001 | 0.43 (0.40,0.46) | <0.001 |
| | 80+ | 0.14 (0.12,0.15) | <0.001 | 0.14 (0.12,0.15) | <0.001 |
| Race (Ref: White) | Black | 0.72 (0.64,0.80) | <0.001 | 0.64 (0.58,0.72) | <0.001 |
| | Other | 0.83 (0.71,0.97) | 0.02 | 0.79 (0.67,0.93) | 0.003 |
| Treated at Teaching Hospital | | 1.13 (1.05,1.21) | <0.001 | 1.07 (0.99,1.15) | 0.09 |
| Visited 2 or More Medical Oncologists (Ref: 1) | | 1.28 (1.22,1.35) | <0.001 | 1.12 (1.06,1.19) | <0.001 |
| Rural (Ref: Urban) | | 1.02 (0.95,1.10) | 0.51 | 0.97 (0.90,1.04) | 0.35 |
| Area Poverty | ≥ 20% | 1.03 (0.94,1.14) | 0.50 | 1.05 (0.95,1.17) | 0.34 |
| Primary Medical Oncologist Predictors | | | | | |
| Gender (Ref: Male) | Female | 1.14 (1.06,1.23) | 0.001 | 1.11 (1.03,1.20) | 0.01 |
| Patient Volume (Ref: 1-4) | 5-9 | 1.30 (1.09,1.55) | 0.003 | 1.33 (1.10,1.60) | 0.002 |
| | 10-19 | 1.40 (1.19,1.66) | <0.001 | 1.39 (1.17,1.66) | <0.001 |
| | 20+ | 1.27 (1.08,1.49) | 0.004 | 1.20 (1.00,1.44) | 0.05 |
| Number of Patient-Sharing Ties to Medical Oncologists | | 1.00 (1.00,1.01) | 0.67 | 0.99 (0.98,1.00) | 0.02 |
| Number of Patient-Sharing Ties to Surgeons | | 1.01 (1.00,1.02) | 0.03 | 1.02 (1.00,1.03) | 0.01 |
| Region (Ref: Northeast) | Midwest | 1.12 (0.98,1.28) | 0.08 | 1.13 (0.98,1.29) | 0.09 |
| | South | 1.09 (0.97,1.23) | 0.15 | 1.04 (0.91,1.19) | 0.53 |
| | West | 1.01 (0.88,1.17) | 0.84 | 0.95 (0.81,1.12) | 0.56 |
| HRR Predictors | | | | | |
| % with Medicare Advantage | Top 50$^{th}$ Percentile | 0.91 (0.84,0.99) | 0.03 | 0.92 (0.84,1.01) | 0.06 |
| % Black | Top Quintile | 1.06 (0.96,1.16) | 0.27 | 1.04 (0.93,1.16) | 0.51 |
| % Hispanic | Top Quintile | 1.03 (0.93,1.14) | 0.54 | 1.10 (0.98,1.24) | 0.11 |
| % Other Race | Top Quintile | 0.99 (0.90,1.09) | 0.79 | 0.99 (0.88,1.10) | 0.80 |
| Physicians per capita | Bottom Quintile | 0.95 (0.84,1.08) | 0.43 | 0.95 (0.83,1.09) | 0.45 |
| Medical Oncologists per capita | Bottom Quintile | 0.95 (0.84,1.07) | 0.36 | 0.99 (0.86,1.14) | 0.88 |

[a] Mixed-effects logistic regression, Random effects for primary medical oncologist and HRR

Abbreviations: CI = confidence interval, HRR = hospital referral region, OR = odds ratio

**Supporting Table 5**. Adjusted Medical Oncologist-Level Models of Oncotype DX Adoption Stratified by Patient Volume, 2008-2009 [a]

| | | 1-4 Patients N=2,680 | | 5-9 Patients N=1,805 | | 10-19 Patients N=2,512 | | 20+ Patients N=2,098 | |
|---|---|---|---|---|---|---|---|---|---|
| | | Adjusted OR (95% CI) | P | Adjusted OR (95% CI) | P | Adjusted OR (95% CI) | P | Adjusted OR (95% CI) | P |
| Gender (Ref: Male) | Female | 1.02 (0.78,1.32) | 0.91 | 1.57 (1.25,1.97) | <0.001 | 1.19 (0.98,1.45) | 0.07 | 1.78 (1.40,2.26) | <0.001 |
| Number of Patient-Sharing Ties to Medical Oncologists | | 0.99 (0.93,1.05) | 0.64 | 0.95 (0.92,0.98) | 0.003 | 0.95 (0.92,0.97) | <0.001 | 0.94 (0.92,0.96) | <0.001 |
| Number of Patient-Sharing Ties to Surgeons | | 1.69 (1.45,1.98) | <0.001 | 1.16 (1.08,1.24) | <0.001 | 1.11 (1.07,1.15) | <0.001 | 1.09 (1.05,1.12) | <0.001 |
| Region (Ref: Northeast) | Midwest | 1.07 (0.76,1.51) | 0.69 | 1.41 (1.04,1.89) | 0.02 | 1.05 (0.81,1.36) | 0.73 | 1.01 (0.70,1.45) | 0.97 |
| | South | 0.82 (0.60,1.14) | 0.23 | 1.11 (0.84,1.46) | 0.47 | 1.23 (0.95,1.59) | 0.11 | 0.91 (0.64,1.28) | 0.57 |
| | West | 1.46 (1.01,2.13) | 0.04 | 0.94 (0.68,1.30) | 0.69 | 0.94 (0.70,1.27) | 0.69 | 0.85 (0.56,1.29) | 0.45 |
| Rural (Ref: Urban) | | 0.73 (0.51,1.06) | 0.09 | 0.93 (0.72,1.21) | 0.60 | 0.97 (0.78,1.21) | 0.76 | 0.96 (0.72,1.28) | 0.78 |
| Area Poverty | ≥ 20% | 0.93 (0.61,1.42) | 0.73 | 1.09 (0.76,1.56) | 0.64 | 1.04 (0.75,1.43) | 0.83 | 0.95 (0.60,1.49) | 0.81 |
| Hospital Referral Region | | | | | | | | | |
| % with Medicare Advantage | Top 50th Percentile | 0.86 (0.66,1.11) | 0.23 | 0.94 (0.76,1.17) | 0.60 | 1.11 (0.92,1.34) | 0.28 | 0.83 (0.65,1.07) | 0.14 |
| % Black | Top Quintile | 1.33 (1.00,1.77) | 0.05 | 0.95 (0.73,1.24) | 0.72 | 0.88 (0.69,1.10) | 0.25 | 1.10 (0.81,1.49) | 0.53 |
| % Hispanic | Top Quintile | 0.93 (0.69,1.25) | 0.60 | 0.97 (0.74,1.26) | 0.79 | 1.08 (0.85,1.38) | 0.53 | 1.09 (0.78,1.54) | 0.60 |
| % Other Race | Top Quintile | 0.92 (0.69,1.23) | 0.56 | 0.95 (0.73,1.23) | 0.70 | 1.02 (0.80,1.29) | 0.89 | 0.97 (0.71,1.33) | 0.83 |
| Physicians per capita | Bottom Quintile | 1.34 (0.86,2.10) | 0.19 | 1.06 (0.74,1.53) | 0.74 | 0.86 (0.64,1.16) | 0.32 | 1.48 (0.99,2.19) | 0.05 |
| Medical Oncologists per capita | Bottom Quintile | 0.96 (0.58,1.60) | 0.87 | 1.03 (0.69,1.53) | 0.90 | 0.88 (0.65,1.19) | 0.38 | 0.90 (0.63,1.29) | 0.56 |

[a] Mixed-effects logistic regression of adoption by the end of 2009, Random effect for HRR

Abbreviations: CI = confidence interval, HRR = hospital referral region, OR = odds ratio

**Supporting Table 6**. Adjusted Medical Oncologist-Level Models of Oncotype DX Adoption Stratified by Patient Volume, 2010-2011 [a]

| | | 1-4 Patients N=1,395 | | 5-9 Patients N=926 | | 10-19 Patients N=883 | | 20+ Patients N=561 | |
|---|---|---|---|---|---|---|---|---|---|
| | | Adjusted OR (95% CI) | P | Adjusted OR (95% CI) | P | Adjusted OR (95% CI) | P | Adjusted OR (95% CI) | P |
| Gender (Ref: Male) | Female | 0.79 (0.52,1.20) | 0.26 | 0.96 (0.69,1.33) | 0.79 | 1.73 (1.22,2.45) | 0.002 | 1.05 (0.69,1.58) | 0.83 |
| Number of Patient-Sharing Ties to Medical Oncologists | | 1.02 (0.97,1.07) | 0.46 | 0.97 (0.93,1.00) | 0.06 | 0.97 (0.94,1.01) | 0.09 | 0.95 (0.92,0.97) | <0.001 |
| Number of Patient-Sharing Ties to Surgeons | | 1.17 (0.90,1.52) | 0.24 | 1.15 (1.04,1.28) | 0.01 | 1.11 (1.02,1.19) | 0.009 | 1.08 (1.01,1.15) | 0.02 |
| Connection to Early Adopter Medical Oncologist (Ref: None) | Co-Location | 2.12 (1.24,3.62) | 0.01 | 0.91 (0.55,1.51) | 0.71 | 0.66 (0.34,1.28) | 0.21 | 1.65 (0.58,4.72) | 0.34 |
| | Patient-Sharing | 0.86 (0.26,2.82) | 0.80 | 0.60 (0.22,1.68) | 0.32 | 0.59 (0.21,1.67) | 0.31 | 1.74 (0.34,8.93) | 0.50 |
| | Both | 2.85 (1.50,5.42) | 0.001 | 0.88 (0.51,1.52) | 0.64 | 0.52 (0.27,1.03) | 0.06 | 1.07 (0.38,3.00) | 0.89 |
| Connection to Early Adopter Surgeon (Ref: None) | Co-Location | 1.18 (0.73,1.90) | 0.50 | 1.44 (0.95,2.18) | 0.08 | 1.23 (0.80,1.88) | 0.34 | 0.81 (0.42,1.56) | 0.52 |
| | Patient-Sharing | 3.86 (1.08,13.86) | 0.03 | 4.07 (1.03,16.06) | 0.04 | 0.81 (0.25,2.65) | 0.72 | 0.60 (0.07,4.95) | 0.62 |
| | Both | 1.83 (0.88,3.81) | 0.10 | 1.73 (0.99,3.03) | 0.05 | 0.91 (0.55,1.52) | 0.72 | 0.76 (0.38,1.52) | 0.44 |
| Region (Ref: Northeast) | Midwest | 0.78 (0.47,1.30) | 0.33 | 0.92 (0.60,1.40) | 0.69 | 1.07 (0.65,1.74) | 0.79 | 1.23 (0.67,2.27) | 0.50 |
| | South | 0.88 (0.57,1.35) | 0.55 | 0.98 (0.67,1.45) | 0.93 | 1.17 (0.74,1.87) | 0.49 | 1.40 (0.78,2.52) | 0.25 |
| | West | 1.08 (0.62,1.87) | 0.79 | 0.97 (0.61,1.55) | 0.91 | 0.68 (0.39,1.18) | 0.16 | 1.13 (0.58,2.24) | 0.71 |
| Rural (Ref: Urban) | | 1.24 (0.76,2.03) | 0.38 | 0.94 (0.63,1.39) | 0.75 | 1.23 (0.84,1.79) | 0.27 | 1.14 (0.69,1.86) | 0.60 |
| Area Poverty | ≥ 20% | 0.81 (0.42,1.57) | 0.52 | 1.25 (0.74,2.13) | 0.40 | 0.77 (0.44,1.33) | 0.33 | 1.69 (0.77,3.73) | 0.19 |
| <u>Hospital Referral Region</u> | | | | | | | | | |
| % with Medicare Advantage | Top 50th Percentile | 1.24 (0.85,1.80) | 0.25 | 0.76 (0.56,1.04) | 0.08 | 0.95 (0.68,1.34) | 0.79 | 0.86 (0.56,1.31) | 0.46 |
| % Black | Top Quintile | 1.51 (1.00,2.29) | 0.05 | 1.05 (0.73,1.51) | 0.80 | 0.88 (0.57,1.34) | 0.53 | 0.83 (0.51,1.34) | 0.43 |
| % Hispanic | Top Quintile | 1.00 (0.65,1.53) | 0.99 | 0.93 (0.62,1.38) | 0.70 | 1.10 (0.69,1.75) | 0.70 | 0.80 (0.46,1.41) | 0.44 |
| % Other Race | Top Quintile | 0.71 (0.46,1.10) | 0.12 | 1.21 (0.84,1.75) | 0.29 | 1.12 (0.72,1.75) | 0.62 | 1.06 (0.63,1.77) | 0.84 |
| Physicians per capita | Bottom Quintile | 0.65 (0.34,1.25) | 0.19 | 0.74 (0.43,1.29) | 0.28 | 0.62 (0.35,1.08) | 0.08 | 0.56 (0.29,1.08) | 0.08 |
| Medical Oncologists per capita | Bottom Quintile | 1.38 (0.70,2.70) | 0.34 | 0.97 (0.54,1.73) | 0.91 | 0.89 (0.51,1.55) | 0.67 | 1.18 (0.64,2.20) | 0.58 |

[a] Mixed-effects logistic regression of adoption by the end of 2011, Random effect for HRR

Abbreviations: CI = confidence interval, HRR = hospital referral region, OR = odds ratio
